# Non-alcoholic fatty liver disease, liver biomarkers and stroke risk: The Reasons for Geographic and Racial Differences in Stroke cohort

**DOI:** 10.1371/journal.pone.0194153

**Published:** 2018-03-12

**Authors:** Kristine S. Alexander, Neil A. Zakai, Steven D. Lidofsky, Peter W. Callas, Suzanne E. Judd, Russell P. Tracy, Mary Cushman

**Affiliations:** 1 Department of Medicine, Larner College of Medicine at the University of Vermont, Burlington, Vermont, United States of America; 2 Department of Pathology and Laboratory Medicine, Larner College of Medicine at the University of Vermont, Burlington, Vermont, United States of America; 3 Department of Mathematics and Statistics, University of Vermont, Burlington, Vermont, United States of America; 4 Department of Biostatistics, School of Public Health, University of Alabama at Birmingham, Birmingham, Alabama, United States of America; 5 Department of Biochemistry, Larner College of Medicine at the University of Vermont, Burlington, Vermont, United States of America; Medizinische Fakultat der RWTH Aachen, GERMANY

## Abstract

**Background and purpose:**

Liver disease, particularly non-alcoholic fatty liver disease (NAFLD), is a risk factor for cardiovascular disease, but little is known about its relationship with ischemic stroke.

**Methods:**

In the Reasons for Geographic and Racial Differences in Stroke (REGARDS) cohort of 30,239 American black and white adults, we assessed baseline NAFLD as fatty liver index (FLI) >60, and assessed liver biomarkers aspartate aminotransferase (AST), alanine aminotransferase (ALT), γ-glutamyl transpeptidase (GGT), and the AST/ALT ratio and risk of incident ischemic stroke over 5.8 years using a case-cohort study design.

**Results:**

Considering 572 strokes and a 1,017-person cohort sample, NAFLD was inversely associated with stroke risk in men (HR: 0.50; 95% CI: 0.26, 0.96), as was being in the highest ALT quintile versus the lowest (HR: 0.39; 95% CI: 0.19, 0.78) and the highest versus lowest GGT quintile (HR: 0.45, 95% CI: 0.24, 0.85), but not in women. Conversely, FLI score above the 90^th^ percentile was associated with increased stroke risk among women (HR: 2.26; 95% CI: 1.14–4.47), but not men. AST was not associated with stroke risk in either sex. AST/ALT ratio >2 was strongly associated with increased stroke risk in whites, but not blacks (HRs: 3.64; 95% CI: 1.42–9.35 and 0.97; 95% CI: 0.45–1.99, respectively; p for interaction = 0.03).

**Conclusions:**

The relationships between NAFLD, liver biomarkers, and ischemic stroke are complex, and sex and race differences we observed require further study and confirmation.

## Introduction

Liver dysfunction can contribute to thrombotic cardiovascular disease (CVD) through effects on synthesis of lipoproteins [1], coagulation proteins [2], and inflammation factors [3]. Many chronic liver diseases, such as hepatitis C and alcoholic liver disease are associated with increased cardiovascular risk [4, 5]. Non-alcoholic fatty liver disease (NAFLD), which is the accumulation of fat in the liver not caused by alcohol use, affects 10–46% of people in the United States [6], and is particularly prevalent in people with type 2 diabetes and obesity. It is considered the “hepatic manifestation of the metabolic syndrome” [7], and is associated with an increased risk of CVD [8–10].

Evaluating the association of liver dysfunction with CVD is challenging as there is no standard measure of liver function, and diagnosis of hepatocyte injury, structural liver disease, and hepatic dysfunction require a clinical diagnosis based on signs, symptoms, and biochemical measures of liver dysfunction. Levels of ALT, AST, and GGT are often increased in NAFLD and other liver diseases, and each has been associated with cardiovascular events including stroke in some cases [11–16]. Three prospective studies specifically investigated the association of imaging-defined NAFLD with stroke [9, 17, 18], with a meta-analysis of these, including only 100 strokes in total, reporting a 2-fold increased risk for stroke with NAFLD (95% CI: 1.46–2.98, p < 0.001) [19].

To extend literature on associations of liver biomarkers and NAFLD with stroke, we assessed the relationship between NAFLD, hepatic biomarkers and incident ischemic stroke in the REasons for Geographic and Racial Differences in Stroke (REGARDS) cohort. We assessed the biomarkers ALT, AST, GGT, the AST/ALT ratio, and the fatty liver index (FLI) [20] as a surrogate marker for NAFLD. We hypothesized that these biomarkers would be associated with increased risk of stroke, and that this risk might differ by race and by sex.

## Methods

REGARDS is a population-based cohort study of 30,239 individuals aged 45 and above, designed to investigate racial and regional disparities in stroke in the contiguous United States [21]. The cohort was recruited from 2003–7. Participants were excluded from enrollment if their medical conditions precluded the expectation of long term participation. By design, 55% of the participants were female, 42% were black, and 56% lived in the stroke belt of the southeastern United States (North Carolina, South Carolina, Georgia, Tennessee, Mississippi, Alabama, Louisiana, and Arkansas). At an initial telephone interview, participants gave verbal informed consent, during which demographic, socioeconomic, and medical history information was collected. At a subsequent at home visit, we obtained written informed consent, blood samples, anthropomorphic measurements, blood pressure, electrocardiogram (ECG), and medication inventory. All study procedures were reviewed and approved by the institutional review boards of the collaborating institutions (University of Alabama at Birmingham Institutional Review Board, University of Vermont Committee on Human Research, University of Cincinnati Institutional Review Board, Wake Forest University Institutional Review Board).

### Study design

**Fig 1:**
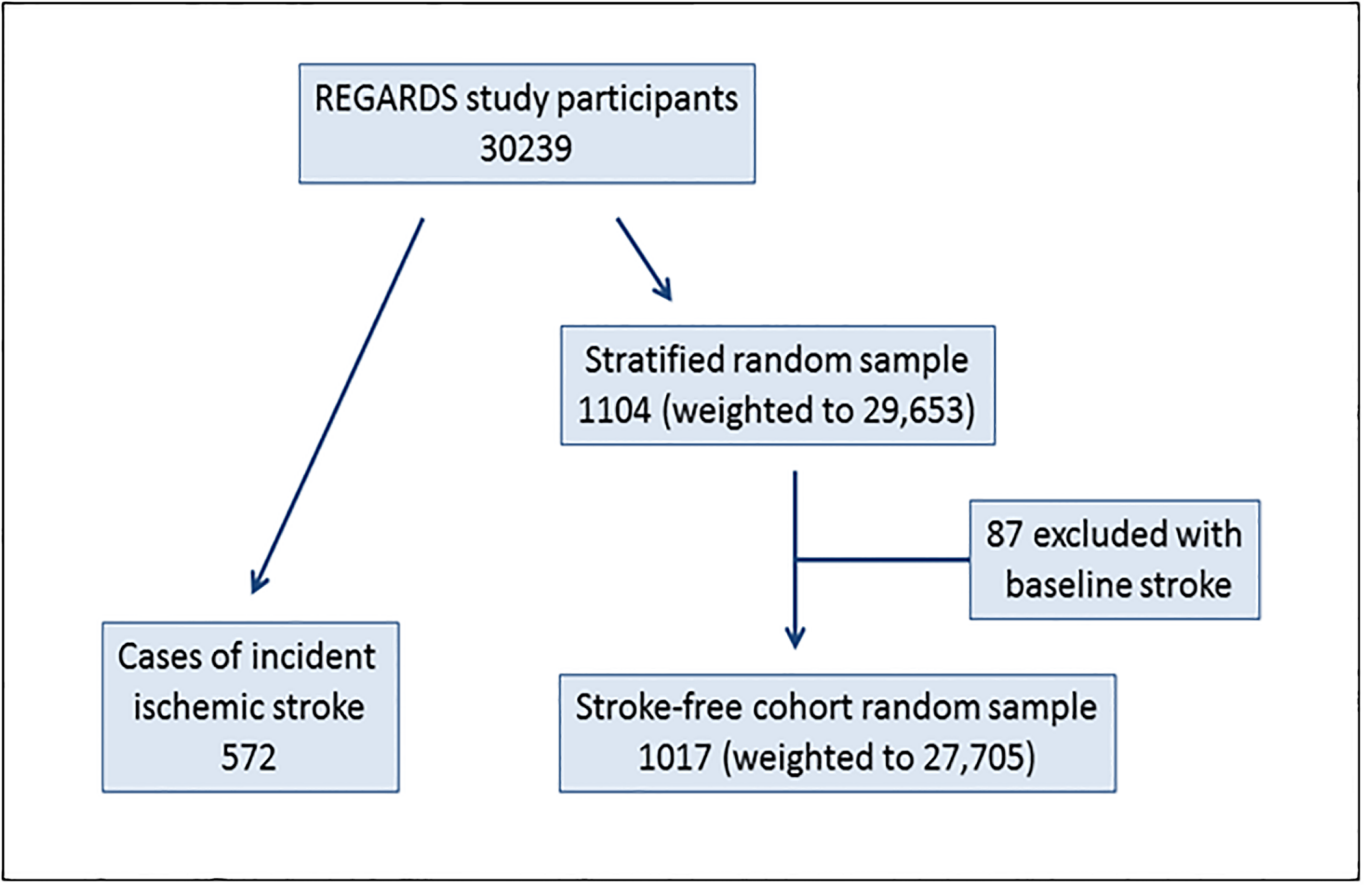
Case-cohort study design.

To minimize assay cost and improve efficiency, we conducted a case-cohort study within REGARDS consisting of 572 cases of incident ischemic stroke and a stratified cohort random sample (CRS) of 1,104 participants (Fig 1), as previously described [22]. Briefly, cases were ascertained through September 1, 2011, with a median follow-up time of 5.8 years. REGARDS participants or their proxies were telephoned every 6 months for health status updates. In cases of death, occurrence of stroke symptoms, or suspected cerebrovascular event, medical records were obtained and reviewed. Strokes events were validated by ≥2 physicians, and classified as hemorrhagic or ischemic. Selection of the cohort sample was stratified on age, race and sex. We excluded 87 CRS participants who had prebaseline stroke.

### Covariates

Race, prebaseline stroke, and alcohol drinks per week were established by participant self-report. Data on statin and warfarin use were collected by self-report. Hypertension was defined as systolic blood pressure ≥140 mmHg or diastolic blood pressure ≥90 mmHg or self-reported use of anti-hypertensive medications. Diabetes was defined as fasting glucose ≥126 mg/dL, non-fasting glucose ≥200mg/dL, self-reported physician diagnosis of diabetes, or self-reported use of diabetes medication. Dyslipidemia was defined as total cholesterol ≥240 mg/dL, low density lipoprotein ≥160 mg/dL, high density lipoprotein ≤40 mg/dL, or use of cholesterol-lowering medications. Atrial fibrillation was established by self-report or presence on baseline electrocardiogram. Left ventricular hypertrophy was determined by baseline electrocardiogram [23]. Baseline CVD was defined as electrocardiogram evidence of myocardial infarction or self-reports of myocardial infarction, coronary artery bypass, stent, angioplasty, or peripheral artery disease.

### Laboratory methods

Fasting blood samples drawn at in-home visits were centrifuged locally and shipped on ice overnight to the study laboratory at the University of Vermont, where they were recentrifuged and stored at -80°C [24]. AST, ALT, and GGT were measured in serum using the Roche Elecsys 2010 analyzer (Roche Diagnostics Indianapolis, IN). Laboratory analytical inter-assay CV ranges were 1.0%-5.4% for ALT, 1.8%-6.8% for AST, and 0.7%-3.0% for GGT.

### Fatty liver index

NALFD status was determined using the FLI, a surrogate marker for non-alcoholic fatty liver disease developed by Bedogni *et*. *al*.[20]. The FLI was designed for use in studies where diagnosis by biopsy or imaging modalities is not feasible. It compares well with ultrasound [25–27] and proton magnetic resonance spectroscopy [28] determinations of hepatic steatosis, though it does not quantitatively predict liver fat. FLI is calculated from body mass index (BMI), GGT, waist circumference, and triglycerides according to the formula:

$$\frac{e^{0.953*\text{log}(\text{triglycerides})+0.139*\text{BMI}+0.718*\text{log}(\text{GGT})+0.053*\text{waist}\;\text{circumference}-15.745}}{1+e^{0.953*\text{log}(\text{triglycerides})+0.139*\text{BMI}+0.718*\text{log}(\text{GGT})+0.053*\text{waist}\;\text{circumference}-15.745}}\times 100$$

Based on previously established cutpoints [29], we defined likely NAFLD as FLI >60, while a score of <20 ruled out NAFLD, and scores of 20 to 60 were indeterminate. Compared to ^1^H-magnetic resonance spectroscopy, an FLI >60 is 91% specific and has a positive likelihood ratio of 5.10, such that individuals with NAFLD are 5 times more likely to score >60, while the negative likelihood ratio for a score <20 is 0.27, indicating that individuals without NAFLD are about 4 times more likely to score below 20 [28]. Individuals who consumed excessive amounts of alcohol (≥14 drinks/week for men, ≥7 drinks/week for women) represented < 5% of the participants, were considered more likely to have alcoholic fatty liver disease than NAFLD, so were excluded from analysis of FLI and stroke risk.

### Statistical analyses

Statistical analyses were performed using SAS 9.3 (Cary, NC). Demographic and stroke risk factor correlates of FLI categories and liver enzyme quintiles were assessed by univariate analyses using the Rao-Scott χ^2^ statistic for categorical variables or linear regression for continuous variables, with weighting to account for stratified sample selection.

Hazard ratios for liver markers and ischemic stroke risk were calculated using weighted Cox proportional hazards models, with 95% confidence intervals (CI) calculated with robust sandwich estimators [30]. Model 1 either adjusted for or was stratified by age, sex, and race. All models also adjusted for an age by race interaction term, due to the known interaction of these factors for stroke risk [31]. Model 2 was additionally adjusted for the Framingham stroke risk factors: systolic blood pressure (SBP), left ventricular hypertrophy (LVH), smoking, prevalent CVD, atrial fibrillation, diabetes, and hypertension medication use. We did not adjust for alcohol use as it was not associated with stroke risk (data not shown).

Primary analysis of FLI was based on the cut points above for fatty liver (<20 and >60), with secondary analyses using FLI as a continuous variable and dichotomized at the 90^th^ percentile. We chose the 90^th^ percentile to identify individuals with a high likelihood of hepatic steatosis [20]. Levels of AST, ALT, and GGT were analyzed in sex-specific quintiles, based on their distribution in the CRS, and as continuous variables, with log-transformation for the latter to correct for skewed distributions. We also assessed AST/ALT ratio as a risk factor, since ratios >2 are associated with alcoholic hepatitis and predict long-term complications from chronic liver disease [32]. Interactions of the liver markers with race and sex were tested using cross-product terms with p<0.10 for the interaction term considered significant.

## Results

In the CRS the mean age was 64.7 years, 55% were women, 41% were black and 39% had a BMI >30 kg/m^2^. Among cases and the CRS, FLI was missing in 133 participants, AST in 117, ALT in 111, and GGT in 100. In Table 1, NAFLD was present in 44% of the CRS based on FLI criteria and only 19% had a low FLI (<20). There was no difference in NAFLD by region of residence or alcohol use, and the strongest correlates of NAFLD were male sex, components of the metabolic syndrome, and LVH, which was 5 times more prevalent in those with NAFLD (Table 1).

**Table 1:** Baseline characteristics by FLI score in the cohort random sample.

	Low FLI (<20)	Intermediate FLI (20–60)	High FLI (>60)	p*	FLI ≥90^th^ Percentile (≥91.4)	p †
**Participants**	19%	36%	44%			
**Age, mean (SD)**	64.5 (11)	66.1 (9)	64.0 (8)	0.11	61 (8)	<0.001
**Women**	73%	53%	49%	<0.001	56%	0.85
**Black**	35%	38%	46%	0.05	47%	0.34
**Stroke belt**	36%	38%	30%	0.39	33%	0.78
**BMI, mean (SD)**	23.1 (2.5)	27.2 (3.0)	33.7 (5.6)	<0.001	40.0 (6.4)	<0.001
**Waist (cm), mean (SD)**	78 (8)	91 (7)	107 (12)	<0.001	119 (12)	<0.001
**SBP (mmHg), mean (SD)**	121 (15)	126 (16)	131 (17)	<0.001	131 (16)	0.04
**Current smoking**	13%	11%	15%	0.43	15%	0.66
**Hypertension**	37%	55%	68%	<0.001	75%	0.002
**Dyslipidemia**	37%	60%	69%	<0.001	64%	0.24
**CVD**	12%	16%	18%	0.25	12%	0.26
**Diabetes**	8%	14%	34%	<0.001	40%	<0.001
**Atrial fibrillation**	10%	7%	10%	0.51	4%	0.06
**LVH**	2%	8%	10%	0.003	10%	0.43
**Alcohol drinks/wk, mean (SD)**	3.9 (15.8)	2.6 (7.9)	1.7 (4.0)	0.15	1.0 (3.3)	0.006
**Statin use**	23%	34%	35%	0.04	32%	0.86

BMI: body mass index, SBP: systolic blood pressure, CVD: cardiovascular disease, LVH: left ventricular hypertrophy

* p values for differences between the groups, by Rao-Scott χ2 statistic (categorical variables) or linear regression (continuous variables)

^†^ p values for ≥90^th^ percentile compared with all others, by Rao-Scott χ2 statistic (categorical variables) or linear regression (continuous variables)

In the fully adjusted model, NAFLD was associated with reduced stroke risk and there was a significant interaction between NAFLD and sex (p = 0.09), but not race (p = 0.9). In men, NAFLD was inversely associated with stroke risk, but there was no association in women (Table 2). In secondary analyses the same pattern was seen for FLI as a continuous variable. By contrast, women in the top 10% of FLI score were at increased risk of stroke compared to those with lower values, while among men the association remained inverse, but not statistically significantly (and the sex interaction term was not significant). To assure that this inverse association in men was not due to use of vasculoprotective medications in high-risk subjects with NAFLD, we tested the effect of adding baseline statin and warfarin use to the fully-adjusted model, but this did not change the nature of the association (HR for NAFLD in men: 0.43; 95% CI: 0.22, 0.84).

**Table 2:** Hazard ratio (95% confidence interval) of ischemic stroke by FLI*.

Model†	NAFLD (FLI >60) vs FLI <20	FLI >90^th^ Percentile vs Lower	Per 10 unit increment of FLI Score
**Model 1**			
All	1.00 (0.69, 1.43)	**1.59 (1.02, 2.46)**	1.02 (0.97, 1.07)
Men	0.67 (0.39, 1.17)	1.26 (0.69, 2.30)	0.96 (0.90, 1.03)
Women	1.55 (0.98, 2.46)	**2.10 (1.15, 3.85)**	**1.09 (1.02, 1.16)**
p for interaction	0.06	0.47	0.10
**Model 2**			
All	**0.65 (0.43, 1.00)**	1.18 (0.68, 2.06)	0.95 (0.90, 1.01)
Men	**0.50 (0.26, 0.96)**	0.75 (0.35, 1.60)	**0.90 (0.83, 0.97)**
Women	1.03 (0.58, 1.82)	**2.26 (1.14, 4.47)**	1.03 (0.95, 1.12)
p for interaction	0.09	0.11	0.14

* Hazard ratios shown in bold were statistically significant based on the confidence interval

^†^ Model 1: adjusted for age, race, and age*race

Model 2: additionally adjusted for the Framingham stroke risk factors

We then examined the individual liver biomarkers AST, ALT, and GGT by sex-specific quintiles (S1–S3 Tables). Age and race were significantly associated with all three biomarkers. In particular, black participants were more likely to be in the lower quintiles of ALT, but the higher quintiles of GGT. Younger age was associated with higher levels of all three biomarkers. While all of the liver biomarkers were significantly associated with waist circumference and BMI, for AST these were inverse relationships, while ALT and GGT had positive associations. Higher GGT and ALT, but not AST, were associated with higher alcohol consumption. High GGT was associated with several other cardiovascular risk factors, including SBP, current smoking, dyslipidemia and LVH. Associations were also seen between ALT and atrial fibrillation, and between AST and diabetes.

**Fig 2:**
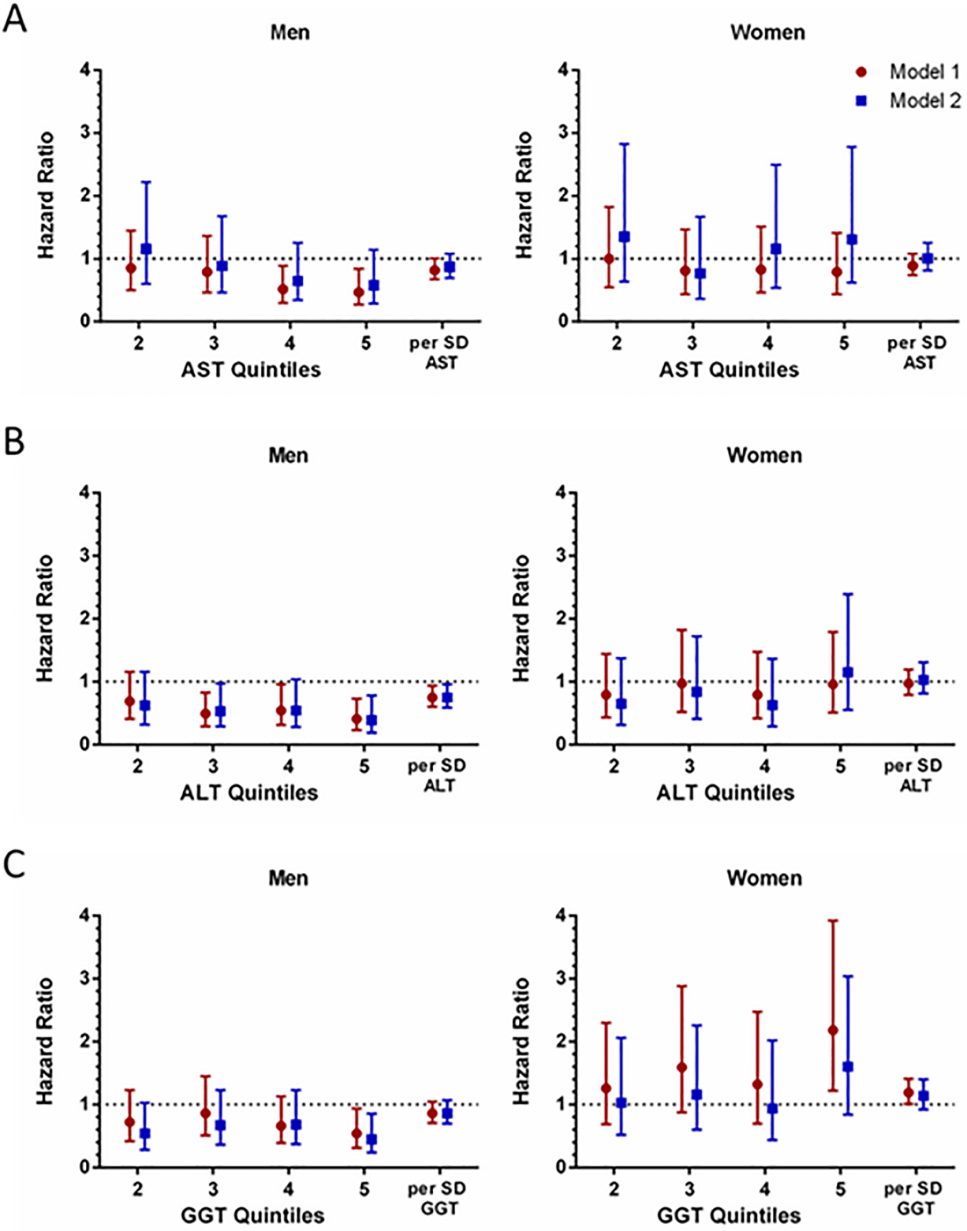
Hazard ratio of ischemic stroke by (A) AST, (B) ALT, and (C) GGT. Model 1: Cox proportional hazards model adjusted for age, race, and age*race Model 2: Additionally adjusted for the Framingham stroke risk factors Percentile cut-off values (U/L) for 20^th^, 40^th^, 60^th^, 80^th^ for men: AST (15.4, 18.5, 21.3, 25.5), ALT (12.1, 15.1, 18.9, 24.2), GGT (17.0 21.6, 28.1, 41.8). For women: AST (14.1, 16.4, 19.4, 23.1), ALT (9.3, 12.5, 14.9, 19.2), GGT (12.8, 16.9, 22.6, 31.2).

Fig 2 shows the hazard ratio of stroke by quintiles of each biomarker, compared with the 1^st^ quintile, and the hazard ratio per SD of each log-transformed marker. Individuals with an AST/ALT ratio > 2 were excluded from these analyses, as this can be an indicator of alcoholic liver disease. GGT quintiles, but not AST or ALT quintiles, showed significant interaction with sex as continuous variables in the fully adjusted model (p = 0.06, 0.53, and 0.41, respectively), so we performed separate analyses for men and women. There were no significant interactions by race.

Higher AST showed a borderline inverse association with stroke risk for men in model 1, but this was not significant after adjustment for stroke risk factors, and there was no association of AST with stroke risk in women. There was a strong inverse association of ALT with ischemic stroke in men, but no association in women in models 1 and 2. For women, higher GGT was associated with stroke in model 1 only, while for men, higher GGT was associated with reduced stroke risk in the fully adjusted model (HR: 0.45 for top quintile; 95% CI: 0.24–0.85).

When the liver enzymes were analyzed as continuous variables (Fig 1), higher ALT again emerged as a marker of lower stroke risk in men (HR: 0.75 per SD; 95% CI: 0.60–0.94), but not in women (HR: 1.03 per SD; 95% CI: 0.81–1.31), after adjustment for stroke risk factors (model 2). GGT, analyzed as a continuous variable, was associated with stroke in women in model 1 (HR: 1.19 per SD; 95% CI: 1.01–1.41), but it was not significantly associated with stroke in men or women after adjustment for stroke risk factors.

An AST/ALT ratio >2 was associated with lower alcohol intake in univariate analysis (S2 Table), but this was not significant after adjustment for age, sex, and race (p = 0.8). Blacks were more likely to have a ratio >2 than whites. Unlike the other measures, there was no sex interaction of AST/ALT >2 with stroke (p = 0.3), but there was a significant race interaction in the fully adjusted model (p = 0.03), so analyses were stratified by race. A ratio >2 was significantly associated with risk of stroke in whites but not blacks, with hazard ratios of 2.74 (95% CI: 1.37–5.48) for whites and 1.29 (95% CI: 0.70–2.40) for blacks, adjusted for age and sex. Adding the Framingham stroke risk factors increased the point estimate in whites but not blacks (HRs: 3.64; 95% CI, 1.42–9.35 and 0.97; 95% CI: 0.47–1.99 in whites and blacks, respectively). In a sensitivity analysis excluding heavy drinkers, results were similar (HRs: 3.64 for whites and 0.95 for blacks).

## Discussion

Our study revealed complex associations between liver biomarkers and stroke risk, with differences by race and sex. NAFLD by FLI and higher ALT and GGT were inversely associated with stroke risk in men, while higher GGT and FLI ≥90^th^ percentile were positively associated with stroke risk among women. An AST/ALT ratio >2, often linked to alcoholic liver disease, was not associated with greater self-reported alcohol use in this cohort, and its relationship with stroke risk differed by race, but not sex, with a strong positive association with stroke in whites but not blacks.

Inverse associations of NAFLD and other liver biomarkers with stroke risk were unexpected, as NAFLD is closely associated with many CVD risk factors including obesity, hypertension, dyslipidemia, and insulin resistance, and it is linked to atherosclerosis progression [4]. Of note, one study addressing risk of pooled cardiovascular endpoints including stroke suggested an inverse association of NAFLD assessed using FLI in persons older than age 50, and a positive association in younger people [33], which is consistent with our findings from our predominantly older cohort. In that study, stroke was not separately analyzed. It is also possible that FLI didn’t adequately detect NAFLD in our study compared to imaging based classification used in other studies [19]. However, the FLI has been linked as might be predicted to risk of incident hypertension [34] and diabetes [35] by others. Despite inverse associations of NAFLD with stroke risk, we also observed positive associations of NAFLD with cardiovascular risk factors using the FLI classification we employed.

The current study is the largest by far that we are aware of to address NAFLD and ischemic stroke risk. While many studies reported positive associations NAFLD and pooled cardiovascular events [9, 17, 19], only three prior studies investigated ischemic stroke separately, with only 100 stroke events considered in totality [15, 17, 18]. These small studies reported mixed results, they did not all adjust for confounders, yet a meta-analysis of these limited studies reported a 2-fold increased risk [19]. Though ischemic stroke and coronary heart disease have many risk factors in common, the fact that they are distinct entities with differing risk profiles [36] may underlie our findings.

Measures of AST, ALT, and GGT have differing specificity for the presence versus absence of liver disease. The aminotransferases AST and ALT are found in the liver, serum, and other tissues, but are primarily indicators of liver damage, with ALT considered to be a better marker of liver injury and steatosis [37]. GGT is less specific to the liver and has been used as a marker of biliary disease and alcohol intake [38]. While increased ALT is often observed with fatty liver and predicts the development of diabetes and the metabolic syndrome [39], its relationship with stroke remains unclear. Similar to our findings, the British Women’s Heart and Health Study saw no association between ALT and stroke in women [13]. In a prospective study of Korean men, ALT was a risk factor for intracerebral hemorrhage, but not ischemic stroke [40]. A German study found no relationship between ALT and overall stroke in middle-aged men and women, but noted an inverse association with ischemic stroke [41], as we observed for men. Unlike our study, they saw no significant interaction between ALT and sex for stroke risk. Interestingly, a meta-analysis found that low ALT predicted cardiovascular mortality, particularly in older people [42]. The researchers speculated that low ALT may be an indicator of poor nutrition, reduced liver cell turnover, or low skeletal muscle mass.

In contrast to ALT, the data linking GGT and AST with stroke risk is more consistent, though in the case of AST, very little research has been published. While AST may be elevated during acute stroke [43], we are aware of only one prospective study on AST and stroke risk, which showed an association between higher AST and intracerebral hemorrhage in Korean men, but no association with other stroke types [40]. Our finding of no association with ischemic stroke is consistent with this. Several studies reported direct associations of GGT with stroke risk [41, 44–47]. Of note, a study in Japan found that GGT was associated with higher risk of ischemic stroke in women but not in men [48], similar to our results.

One purpose of our study was to investigate racial differences in associations between liver biomarkers and ischemic stroke. In the United States, blacks have a lower prevalence of NAFLD [49], but an increased risk of stroke, especially at younger ages [31]. Although black participants had higher FLI scores and GGT than white participants, we did not see significant differences by race in the relationships of liver markers with stroke risk, with the exception of the AST/ALT ratio >2. An elevated AST/ALT ratio can be an indicator of alcoholic hepatitis and of fibrosis and cirrhosis in chronic liver disease [32], and we found an association with stroke risk in whites only.

The strengths of this study include the large, well-characterized, prospective cohort of geographically dispersed blacks and whites. We relied on physician-adjudicated stroke outcomes to minimize misclassification. The major limitation is the use of a surrogate marker for NAFLD. The gold standard for clinical diagnosis of NAFLD is liver biopsy, which is not appropriate for epidemiological studies. While the FLI compares well with ultrasound determination of fatty liver, it is less accurate than liver biopsy or magnetic resonance imaging for identification and grading of liver steatosis [50]. In support of its use, we saw strong correlations between FLI and components of the metabolic syndrome, and a higher prevalence of NAFLD in men compared to women, similar to findings using imaging-based NAFLD assessment [29, 51]. Also, the association of NAFLD by FLI with stroke showed a similar pattern to that of ALT, which is also used as a surrogate marker for NAFLD. As the FLI has not been validated in a black population, and we observed unexpectedly higher, not lower prevalence of NAFLD in blacks, it is possible that the FLI misclassified NAFLD in blacks. This misclassification would bias results to the null hypothesis yet we observed the same association of NAFLD with stroke risk in blacks and whites, supporting the validity of the FLI in blacks. We did not have information on viral infections to determine any impact on the findings. We were also unable to investigate the role of alcohol use in more depth, due to low self-reported alcohol consumption in this cohort.

## Conclusions

High levels of several of the liver disease biomarkers, especially those related to NAFLD, were associated with lower stroke risk in men, but with higher risk or no association in women. Particularly in the case of GGT and ALT, men in the lowest quintile were at increased stroke risk. Low levels of these biomarkers are not considered to be indicators of any disease process, and it is unclear what this relationship represents. While liver disease is gaining appreciation as a risk factor for atherosclerotic CVD, there is currently little known about differences in the relationship between liver disease and CVD by sex [52] or of the association of liver biomarkers with stroke. Further work is needed to confirm our results and identify the mechanisms and implications of these findings.

## Supporting information

S1 Table Participant characteristics by hepatic biomarker quintiles: Cohort random sample. (DOCX)

S2 Table Participant characteristics by ALT quintiles. (DOCX)

S3 Table Participant characteristics by GGT quintiles. (DOCX)

S4 Table Participant characteristics by AST/ALT: cohort random sample. (DOCX)
