# Supplementary material for: Non-alcoholic fatty liver disease, liver biomarkers and stroke risk: The Reasons for Geographic and Racial Differences in Stroke cohort
Source: PLoS One. 2018 Mar 12;13(3):e0194153. doi: 10.1371/journal.pone.0194153 (PMC5847237; doi:10.1371/journal.pone.0194153)
Supplement: S1 Table — (DOCX) [file pone.0194153.s001.docx]

**S1 Table: Participant characteristics by hepatic biomarker quintiles: cohort random sample**

|  | AST 1^st^ | AST 5^th^ | p* | ALT 1^st^ | ALT 5^th^ | p* | GGT 1^st^ | GGT 5^th^ | p* |
| --- | --- | --- | --- | --- | --- | --- | --- | --- | --- |
| **Age, mean (SD)** | 65.2 (9.9) | 64.2 (8.4) | 0.05 | 66.9 (10.3) | 62.5 (7.9) | <0.001 | 66.0 (10.7) | 62.8 (8.8) | <0.001 |
| **Black** | 45% | 42% | 0.02 | 51% | 40% | 0.03 | 26% | 49% | <0.001 |
| **Stroke belt** | 34% | 33% | 0.60 | 39% | 27% | 0.17 | 37% | 31% | 0.06 |
| **BMI, mean (SD)** | 31.0 (7.0) | 28.1 (5.6) | <0.001 | 29.2 (6.4) | 30.0 (5.4) | <0.001 | 27.5 (4.9) | 30.1 (6.4) | <0.001 |
| **Waist circ. (cm), mean (SD)** | 100 (15.6) | 94 (15.4) | <0.001 | 96 (14.7) | 98 (13.9) | 0.003 | 92 (14.8) | 98 (15.1) | <0.001 |
| **SBP (mmHg), mean (SD)** | 128 (17) | 126 (15) | 0.12 | 130 (19) | 128 (13) | 0.08 | 124 (14) | 128 (16) | 0.001 |
| **Current smoking** | 20% | 13% | 0.17 | 19% | 12% | 0.21 | 7% | 16% | 0.01 |
| **Hypertension** | 52% | 48% | 0.37 | 49% | 47% | 0.26 | 48% | 47% | 0.26 |
| **Dyslipidemia** | 60% | 58% | 0.43 | 58% | 62% | 0.18 | 48% | 65% | 0.04 |
| **CVD** | 20% | 24% | 0.02 | 19% | 16% | 0.08 | 12% | 19% | 0.27 |
| **Diabetes** | 37% | 19% | <0.001 | 28% | 21% | 0.17 | 18% | 28% | 0.21 |
| **Atrial fibrillation** | 9% | 9% | 0.57 | 8% | 4% | 0.04 | 6% | 6% | 0.22 |
| **LVH** | 9% | 7% | 0.32 | 9% | 8% | 0.55 | 5% | 13% | 0.03 |
| **Alcohol, Drinks/wk (SD)** | 1.7 (4.0) | 3.1 (8.5) | 0.26 | 1.3 (3.6) | 2.7 (8.2) | 0.006 | 1.6 (3.9) | 2.7 (5.9) | 0.02 |
| **Statin use** | 28% | 29% | 0.26 | 30% | 38% | 0.18 | 27% | 35% | 0.56 |

* p values for overall difference among the 5 quintiles

Sex-specific quintile cut points shown in Fig 1.
