# Supplementary material for: Non-alcoholic fatty liver disease, liver biomarkers and stroke risk: The Reasons for Geographic and Racial Differences in Stroke cohort
Source: PLoS One. 2018 Mar 12;13(3):e0194153. doi: 10.1371/journal.pone.0194153 (PMC5847237; doi:10.1371/journal.pone.0194153)
Supplement: S2 Table — (DOCX) [file pone.0194153.s002.docx]

**S2 Table. Participant characteristics by ALT quintiles**

|  | **ALT Quintiles** | | | | |  |
| --- | --- | --- | --- | --- | --- | --- |
| **Characteristic, mean (SD) or frequency** | **1**  **(0-10.6 U/L)**  **n = 5,335** | **2**  **(10.6-13.7 U/L)**  **n = 5,620** | **3**  **(13.7-16.3 U/L)**  **n = 5,437** | **4**  **(16.3-21.5 U/L)**  **n = 5,513** | **5**  **(≥21.5 U/L)**  **n = 5,586** | **p** |
| **Sex**  **Women**  **Men** | 75%  25% | 58%  42% | 60%  40% | 44%  56% | 37%  63% | <0.001 |
| **Race**  **Black**  **White** | 55%  45% | 40%  60% | 39%  61% | 39%  61% | 33%  67% | 0.002 |
| **Age** | 67.1 (10.5) | 66.1 (10.1) | 64.7 (8.8) | 64.3 (8.8) | 62.9 (8.2) | <0.001 |
| **Region**  **Stroke belt**  **Buckle**  **Nonbelt** | 38%  19 %  43% | 32%  20%  47% | 40%  14%  46% | 35%  19%  46% | 28%  18%  54% | 0.42 |
| **Hypertension** | 60% | 56% | 60% | 60% | 51% | 0.42 |
| **BMI (kg/m^2^)** | 29.3 (6.8) | 28.4 (5.6) | 29.4 (6.4) | 28.8 (5.5) | 29.7 (5.0) | 0.15 |
| **Waist circumference, cm** | 94 (15) | 94 (16) | 94 (14) | 96 (13) | 99 (14) | <0.001 |
| **Smoking**  **Current**  **Past**  **Never** | 15%  38%  47% | 15%  32%  53% | 13%  39%  48% | 17%  43%  40% | 11%  42%  47% | 0.27 |
| **Dyslipidemia** | 48% | 57% | 62% | 63% | 62% | 0.04 |
| **Diabetes** | 26% | 24% | 13% | 23% | 20% | 0.07 |
| **Baseline CHD** | 14% | 18% | 8% | 23% | 18% | 0.008 |
| **Statin Use** | 30% | 29% | 38% | 28% | 38% | 0.18 |
