# Supplementary material for: Non-alcoholic fatty liver disease, liver biomarkers and stroke risk: The Reasons for Geographic and Racial Differences in Stroke cohort
Source: PLoS One. 2018 Mar 12;13(3):e0194153. doi: 10.1371/journal.pone.0194153 (PMC5847237; doi:10.1371/journal.pone.0194153)
Supplement: S3 Table — (DOCX) [file pone.0194153.s003.docx]

**S3 Table. Participant characteristics by GGT quintiles**

|  | **GGT Quintiles** | | | | |  |
| --- | --- | --- | --- | --- | --- | --- |
| **Characteristic, mean (SD) or frequency** | **1**  **(0-14.4 U/L)**  **n = 5,368** | **2**  **(14.4-19.3 U/L)**  **n = 5,560** | **3**  **(19.3-25.1 U/L)**  **n = 5,559** | **4**  **(25.1-37.8 U/L)**  **n = 5,563** | **5**  **(≥37.8 U/L)**  **n = 5,534** | **p** |
| **Sex**  **Women**  **Men** | 77%  23% | 54%  46% | 57%  43% | 45%  55% | 42%  58% | <0.001 |
| **Race**  **Black**  **White** | 32%  68% | 37%  63% | 42%  58% | 46%  54% | 48%  52% | 0.03 |
| **Age** | 66.2 (10.8) | 65.6 (9.1) | 65.4 (9.1) | 64.3 (9.0) | 63.4 (8.6) | <0.001 |
| **Region**  **Stroke belt**  **Buckle**  **Nonbelt** | 37%  19%  44% | 39%  12%  49% | 38%  19%  43% | 28%  23%  50% | 33%  18%  49% | 0.35 |
| **Hypertension** | 54% | 52% | 61% | 60% | 60% | 0.27 |
| **BMI (kg/m^2^)** | 27.4 (5.2) | 28.9 (6.0) | 29.9 (6.4) | 29.5 (5.4) | 29.9 (6.0) | <0.001 |
| **Waist circumference, cm** | 89 (13.9) | 96 (14.6) | 97 (14.2) | 97 (13.3) | 98 (14.7) | <0.001 |
| **Smoking**  **Current**  **Past**  **Never** | 9%  30%  60% | 11%  44%  45% | 16%  45%  38% | 18%  32%  50% | 15%  41%  44% | 0.005 |
| **Dyslipidemia** | 49% | 57% | 54% | 65% | 67% | 0.01 |
| **Diabetes** | 18% | 21% | 22% | 17% | 28% | 0.24 |
| **Baseline CHD** | 13% | 11% | 21% | 16% | 20% | 0.10 |
| **Statin Use** | 27% | 32% | 34% | 35% | 35% | 0.56 |
