# Supplementary material for: Non-alcoholic fatty liver disease, liver biomarkers and stroke risk: The Reasons for Geographic and Racial Differences in Stroke cohort
Source: PLoS One. 2018 Mar 12;13(3):e0194153. doi: 10.1371/journal.pone.0194153 (PMC5847237; doi:10.1371/journal.pone.0194153)
Supplement: S4 Table — (DOCX) [file pone.0194153.s004.docx]

**S4 Table. Participant characteristics by AST/ALT: cohort random sample**

| **Characteristic, mean (SD) or frequency** | **AST/ALT ≤2** | **AST/ALT >2** | **P** |
| --- | --- | --- | --- |
| **Participants** | 95% | 5% |  |
| **Age** | 64.3 (9.2) | 72.7 (9.3) | <0.001 |
| **Sex**  **Women**  **Men** | 54%  46% | 74%  26% | 0.003 |
| **Race**  **Black**  **White** | 40%  60% | 65%  35% | 0.001 |
| **Region**  **Stroke belt**  **Buckle**  **Nonbelt** | 33%  18%  48% | 28%  25%  47% | 0.56 |
| **BMI (kg/m^2^)** | 29.3 (5.9) | 27.0 (5.1) | <0.001 |
| **Waist circumference, cm** | 96 (15) | 91 (13) | 0.003 |
| **Current Smoker** | 13% | 26% | 0.02 |
| **Hypertension** | 48% | 65% | 0.03 |
| **Dyslipidemia** | 59% | 48% | 0.19 |
| **CVD** | 16% | 25% | 0.14 |
| **Diabetes** | 21% | 22% | 0.89 |
| **Atrial fibrillation** | 9% | 5% | 0.26 |
| **Alcohol drinks/wk, mean (SD)** | 2.5 (9.1) | 1.4 (4.4) | 0.04 |
| **Statin use** | 32% | 40% | 0.31 |
